# Developing evidence-based dentistry skills: how to interpret randomized clinical trials and systematic reviews

**DOI:** 10.1186/s40510-014-0058-5

**Published:** 2014-10-30

**Authors:** Juliana Kiriakou, Nikolaos Pandis, Phoebus Madianos, Argy Polychronopoulou

**Affiliations:** Department of Preventive and Community Dentistry, School of Dentistry, University of Athens, 2 Thivon Str, P.O. Box 18018, Athens, 115 27 Greece; Department of Orthodontics and Dentofacial Orthopedics, School of Dental Medicine/Medical Faculty, University of Bern, Freiburgstrasse 7 CH-3010, Bern, Switzerland; Department of Periodontology, School of Dentistry, University of Athens, 2 Thivon Str, Athens, 115 27 Greece

**Keywords:** Randomized controlled trials, Systematic reviews, Critical appraisal, Evidence-based dentistry

## Abstract

Decision-making based on reliable evidence is more likely to lead to effective and efficient treatments. Evidence-based dentistry was developed, similarly to evidence-based medicine, to help clinicians apply current and valid research findings into their own clinical practice. Interpreting and appraising the literature is fundamental and involves the development of evidence-based dentistry (EBD) skills. Systematic reviews (SRs) of randomized controlled trials (RCTs) are considered to be evidence of the highest level in evaluating the effectiveness of interventions. Furthermore, the assessment of the report of a RCT, as well as a SR, can lead to an estimation of how the study was designed and conducted.

## Review

### Introduction

We live in the age of information, innovation, and change. The number of published studies in the dental literature increases dramatically every year. Clinicians are required to base their decisions on the best available research evidence by critically appraising and incorporating sound scientific evidence into everyday clinical practice [1]. The clinicians' difficulty of staying current can be facilitated by integrating basic skills of evidence-based dentistry (EBD), such as the ability to identify and critically appraise evidence, into everyday practice [2].

#### Defining evidence-based dentistry

The practice of evidence-based dentistry consists in dentists critically applying relevant research findings to the care of patients [3]. The American Dental Association (A.D.A.) defines evidence-based dentistry as ‘an approach to oral health care that requires the judicious integration of systematic assessments of clinically relevant scientific evidence, relating to the patient's oral and medical condition and history, with the dentist's clinical expertise and the patient's treatment needs and preferences.’ Evidence-based dentistry is based on three important domains: the best available scientific evidence, dentist's clinical skills and judgment, and patient's needs and preferences. Only when all three are given due consideration in individual patient care is EBD actually being practiced [4].

#### Why is evidence-based dentistry important?

Practicing evidence-based dentistry reassures the quality improvement of health-care delivery by incorporating effective practices, while eliminating those that are ineffective or inappropriate [5]. The main advantage of EBD is that, in fact, it uses significant findings obtained from large clinical trials and systematic reviews and applies them to the individual patient's needs. In this way, clinicians are able to deliver more focused treatment, while patients receive optimal care [6].

#### The five steps of evidence-based dentistry practice

The practice of EBD involves five essential steps [4,7,8]:

1. Developing a clear, clinically focused question
2. Identifying, summarizing, and synthesizing all relevant studies that directly answer the formulated question
3. Appraising evidence in terms of validity and applicability
4. Combining research evidence with clinical expertise and patients characteristics
5. Assessing the successful implementation of previous steps

#### Choosing the best form of evidence

Some research designs are more effective than others in their ability to answer specific research questions. Rules of evidence have been established to grade evidence according to its strength, giving rise to the concept of ‘hierarchy of evidence.’ The hierarchy provides a framework for rating evidence and indicates which study types should be given more weight when assessing the same question [9]. At the top of the hierarchy, we find high-quality systematic reviews of randomized controlled trials, with or without meta-analysis together with randomized controlled trials (RCTs) of very low risk of bias (Table 1) [10].

**Table 1:** **Revised grading system for recommendations in evidence-based guidelines [** 10 **]**

	**Levels of evidence**
1++	High-quality meta-analyses, systematic reviews of RCTs, or RCTs with a very low risk of bias
1+	Well-conducted meta-analyses, systematic reviews of RCTs, or RCTs with a low risk of bias
1−	Meta-analyses, systematic reviews or RCTs, or RCTs with a high risk of bias
2++	High-quality systematic reviews of case-control or cohort studies or high-quality case-control or cohort studies with a very low risk of confounding, bias, or chance and a high probability that the relationship is causal
2+	Well-conducted case-control or cohort studies with a low risk of confounding, bias, or chance and a moderate probability that the relationship is causal
2−	Case-control or cohort studies with a high risk of confounding, bias, or chance and a significant risk that the relationship is not causal
3	Non-analytic studies, e.g., case reports, case series
4	Expert opinion

#### Classification of studies based on research design

Clinical research can be either observational or experimental. In *observational* studies, the investigator observes patients at a point in time or over time, without intervening. They may be *cross-sectional*, providing a snapshot picture of a population at a particular point in time, or *longitudinal*, following the individuals over a period of time. Observational studies may also be *prospective*, when the data are collected forward in time from the beginning of the study, or *retrospective*, in which the information is obtained by going backwards in time [11,12].

The most common types of observational studies are:

- Cohort studies
  In a cohort study, people are divided into cohorts, based on whether they have been exposed or not to a treatment, and then they are followed over a period of time to note the occurrence of an event, or not [11].
- Case-control studies
  In a case-control study, a sample of cases is compared with a group of controls who do not have the outcome of interest. The cases and controls are then categorized according to whether or not they have been exposed to the risk factor [12].
- Cross-sectional studies
  Cross-sectional studies attempt to investigate an association between a possible risk factor and a condition. The data concerning the exposure to the causal agent and outcome are collected simultaneously [13].
- Case reports and case series
  A case report is a descriptive report of a single patient. A case series is a descriptive report on a series of patients with a condition of interest. No control group is involved [14].
  *Experimental* is the type of study in which the investigator intervenes so as to observe the effect on the outcome being studied [12]. Experimental studies can be either controlled (a comparison group exists) or uncontrolled. Sutherland [11] suggests that *uncontrolled* studies provide very weak evidence and should not be used as a reference for decision-making. Studies with experimental design can be distinguished in those where the comparisons are made between subjects (*parallel groups* design) or within subjects (*matched* design or *cross-over* or split-mouth studies). The *clinical trial* is a particular form of experimental study performed on humans [12]. A well-designed trial involves a comparison between groups formed after the randomization of patients to treatments. This type of study is called a *randomized controlled clinical trial* (RCT) [15].

#### The randomized controlled clinical trial

The randomized controlled clinical trial is a specific type of scientific experiment which is characterized by several distinguishing features. A fundamental feature of a clinical trial is that it is comparative in nature. This means that the results of a group of patients who are receiving the treatment under investigation (treatment group) are compared with those of another group of patients with similar characteristics who are not receiving the particular treatment (*control group*) [15]. In the RCT, the participants are randomly assigned to either experimental or control groups in a way that each participant has an equal probability of being assigned to any given group (*randomization*) [16]. As the two groups are identical apart from the treatment being compared, any differences in outcomes are attributed to the intervention [17].

An essential issue of a RCT is the concealment of the allocation sequence from the investigators who assign participants to treatment groups (*allocation concealment*). Allocation concealment should not be confused, though, with *blinding*, which corresponds to whether patients and investigators know or do not know which treatment they received [18]. Even though the validity of a RCT depends mainly on the randomization procedure, it is almost inevitable that after randomization some participants would not complete the study [19]. *Intention-to-treat* (*ITT*) *analysis* is a strategy that ensures that all patients are included in the analysis as part of the groups they were originally randomized, regardless of whether they withdrew or did not receive the treatment. Application of true ITT analysis requires some assumptions and some form of imputations for the missing data [20,21].

The RCT is the most scientifically rigorous method of hypothesis testing available [20] and is regarded as the gold standard trial for evaluating the effectiveness of interventions [17]. The RCT is the only study which allows investigators to balance unknown prognostic factors at baseline (selection bias), this being its main advantage. Random allocation does not, however, protect RCTs against other types of bias (Table 2) [21]. *Bias* refers to the systematic (not random) deviation from the truth [22].

**Table 2:** **Classification scheme of bias in RCTs [** 22 **]**

**Type of bias**	**Prevention/elimination of bias**
▪ Selection bias	Proper randomization
▪ Performance bias	Blinding of participants and people administering treatment
▪ Detection bias	Blinding of outcome assessors/analyzers
▪ Attrition bias	Blinding participants, intention-to-treat analysis
▪ Publication bias	Trial registration, no selective reporting
▪ Other biases	No selective reporting, meticulous study design

**Figure 1:**
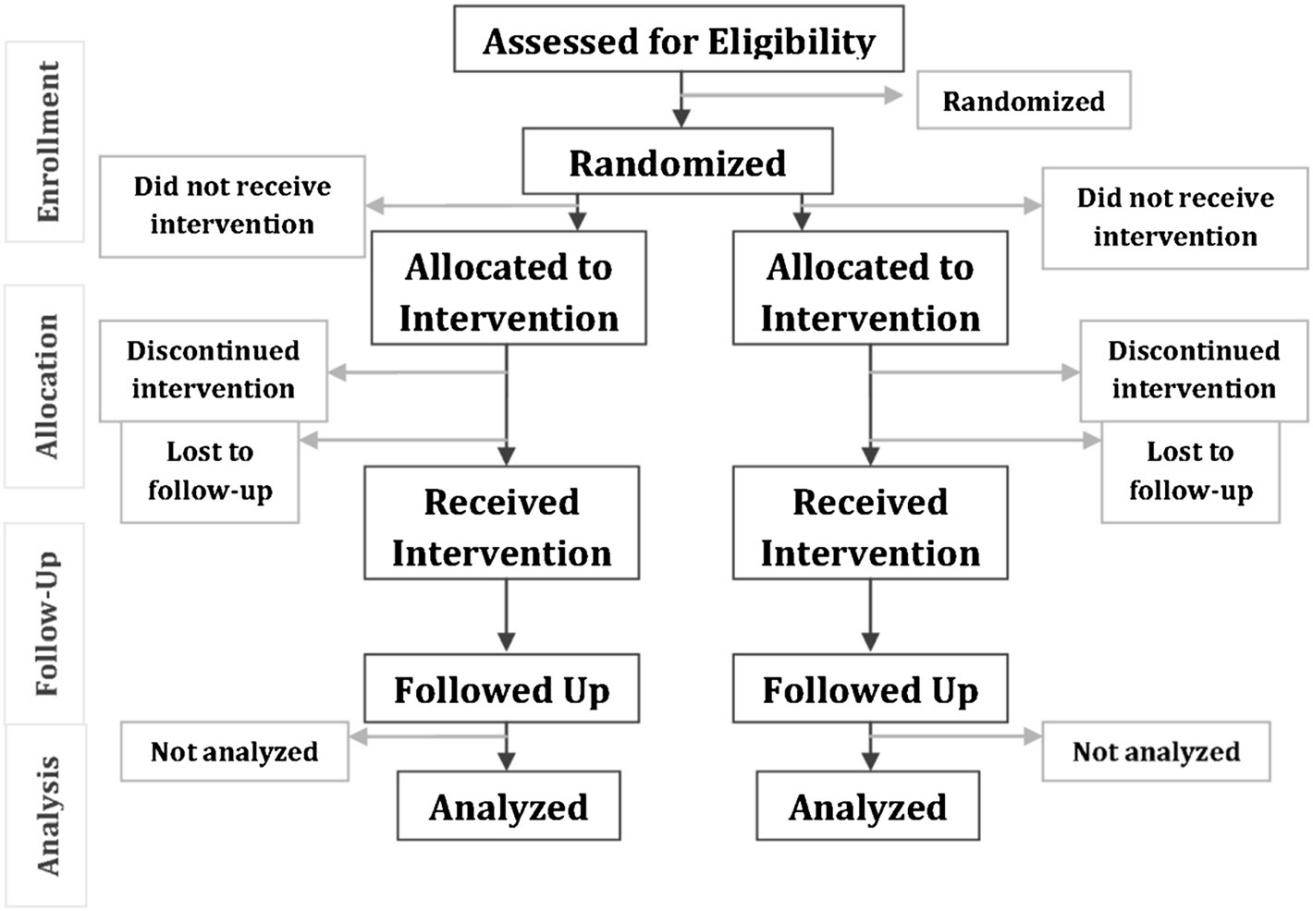
**Flow diagram of the progress through the phases of a parallel RCT of two groups.** Modified from CONSORT 2010 [23].

Most randomized controlled clinical trials have parallel designs in which each group of participants is exposed to only one of the interventions of interest (Figure 1) [23].

#### Understanding systematic reviews

It is well recognized that health-care decisions should not be based merely on one or two studies but take into consideration all the scientific research available on a specific topic [24]. Still, applying research into practice may be time consuming as it is often impractical for readers to track down and review all of the primary studies [2,25]. Systematic reviews are particularly useful in synthesizing studies, even those with conflicting findings [24]. A *systematic review* is a form of research that uses explicit methods to identify comprehensively all studies for a specific focused question, critically appraise them, and synthesize the world literature systematically [26-28]. The explicit methods used in systematic reviews limit bias and, hopefully, will improve reliability and accuracy of conclusions [29].

Developing a systematic review requires a number of discrete steps [22]:

1. Defining an appropriate health-care question
2. Searching the literature
3. Assessing the selected studies
4. Synthesizing the findings
5. Placing the findings in context

##### What are Cochrane reviews?

*Cochrane reviews* are systematic reviews of primary research in human health care and health policy, undertaken by members of The Cochrane Collaboration [30] adhering to a specific methodology [31]. The Cochrane Collaboration is an international organization that aims to organize medical research information in a systematic way promoting the accessibility of systematic reviews of the effects of health-care interventions [31].

##### What is a meta-analysis?

Frequently, data from all studies included in a systematic review are pooled quantitatively and reanalyzed; this is called a *meta-analysis* [32]. This technique increases the size of the ‘overall sample’ and ultimately enhances the statistical power of the analysis [33]. A meta-analysis can be included in a well-executed systematic review. Quantitative synthesis should only be applied under certain conditions such as when the studies to be combined are clinically and statistically homogeneous. If the original review was biased or unsystematic, then the meta-analysis may provide a false measure of treatment effect [34].

#### Principles of critical appraisal

For any clinician, the key to assessing the usefulness of a clinical study and interpreting the results to a particular subject is through the process of critical appraisal. *Critical appraisal* is an essential skill of evidence-based dentistry which involves systematic assessment of research allowing, thus, clinicians to apply valid evidence in an efficient manner. When critically appraising research, there are three main areas that should be assessed: validity, importance, and applicability [35].

A study which is sufficiently free from bias is considered to have *internal validity* [36]. Common types of bias that affect internal validity include allocation bias, blinding, data collection methods, dropouts, etc. [37]. *External validity* refers to whether the study is asking an appropriate research question, as well as whether the study results reflect what can be expected in the population of interest (generalizability) [22].

#### Appraising a randomized controlled trial

Randomized controlled trials are the standard for testing the efficacy of health-care interventions. The better a RCT is designed and conducted, the more likely it is to provide a true estimate of the effect of an intervention. When reading a RCT article, we should focus on certain issues in order to decide whether the results of the study are reliable and applicable to our patient [38].

- The validity of the trial methodology
  During the process of appraising a RCT, readers should evaluate the following:
  1. Clear, appropriate research question
    The clinical question should specify the type of participants, interventions, and outcomes that are of interest [22].
  2. Randomization
  3. Allocation concealment
  4. Blinding
  5. Intention-to-treat analysis
    The concepts of randomization, allocation concealment, blinding, and ITT analysis were previously explained.
  6. Statistical power
    McAlister et al. define the *statistical power* of a RCT as ‘the ability of the study to detect a difference between the groups when such a difference exists’. The size of the sample must be large enough to raise the trial's possibilities of answering the research question and, thus, enhancing the statistical power of the trial [39].
- The magnitude and significance of treatment effect
  *Magnitude* refers to the size of the treatment effect. The *effect size* represents the degree to which the two interventions differ. Effect size in RCTs may be reported in various ways including mean difference, risk ratio, odds ratio, risk difference, rate and hazard ratio, and numbers needed to treat [40,41].
  *Statistical significance* refers to the likelihood that a relationship is caused by something other than simple random chance. Probability (*p*) values and confidence intervals (CI) are used to assess statistical significance [42]. A statistically significant result indicates that there is probably a relationship between the variables of interest, but does not show whether this is important. *Clinical significance* refers to the practical meaning of the effect of an intervention to patients and health-care providers [43].
- Generalizability of trial results
  The fact that evidence addresses the effectiveness of a treatment does not mean that all patients will necessarily benefit from that. Thus, a clinician should always examine the similarities of the study participants with his own patients together with the proportion between the benefits and adverse events of the therapy [38].

##### Tools assessing the quality of RCTs

Many tools have been proposed for assessing the quality of studies. Most tools are scales in which various quality elements are scored or checklists in which specific questions are asked [44]. One method used to critically appraise randomized trials is the *Risk of Bias Tool*, developed by The Cochrane Collaboration, which is based on a critical evaluation of six different domains [22]. The *Critical Appraisal Skills Program* (CASP), also, aims to help people develop the necessary skills to interpret scientific evidence. CASP has released critical appraisal checklists for different types of research studies that cover three main areas of a study: validity, results, and clinical relevance [45]. Likewise, the *Scottish Intercollegiate Guidelines Network* (*SIGN*) has developed a methodology checklist in order to facilitate the assessment of a RCT. This checklist consists of three sections, each covering key topics of a trial [46].

##### Tools assessing the reporting quality of RCTs

To assess a trial accurately, readers need complete and transparent information on its methodology and findings. However, the reporting of RCTs is often incomplete [45-50] aggravating problems arising from poor methodology [51-56]. The Consolidated Standards of Reporting Trials (CONSORT) statement, which is aimed at trials with a parallel design, consists of a checklist of essential items that should be included in the report of a RCT together with a diagram showing the flow of participants through the phases of the trial. The objective of CONSORT is to provide guidance to authors about how to improve the reporting of their trials. Additionally, it can be used by readers, peer reviewers, and editors to help them critically appraise reports of RCTs [23].

Many clinicians base their assessment of a trial, or even their clinical decisions, on the information reported in the abstract, as they only read, or have access to, the abstracts of journal articles [57]. A well-written and constructed abstract should help the readers assess quickly the validity and applicability of the trial findings [58]. Several studies have accentuated the need for improvement in the reporting of conference abstracts and journal abstracts of RCTs [59]. The CONSORT for Abstracts (Table 3) provides a list of essential items that authors should consider when reporting a randomized trial in any journal or conference abstract [60].

**Table 3:** **Items to include when reporting a randomized trial in a journal or conference abstract [** 60 **]**

**Item**	**Description**	**Reported on line number**
Title	Identification of the study as randomized	
Authors^a^	Contact details for the corresponding author	
Trial design	Description of the trial design (e.g., parallel, cluster, non-inferiority)	
Methods		
Participants	Eligibility criteria for participants and the settings where the data were collected	
Interventions	Interventions intended for each group	
Objective	Specific objective or hypothesis	
Outcome	Clearly defined primary outcome for this report	
Randomization	How participants were allocated to interventions	
Blinding (masking)	Whether or not participants, caregivers, and those assessing the outcomes were blinded to group assignment	
Results		
Numbers randomized	Number of participants randomized to each group	
Recruitment	Trial status	
Numbers analyzed	Number of participants analyzed in each group	
Outcome	For the primary outcome, a result for each group and the estimated effect size and its precision	
Harms	Important adverse events or side effects	
Conclusions	General interpretation of the results	
Trial registration	Registration number and name of trial register	
Funding	Source of funding	

^a^This item is specific to conference abstracts.

#### Appraising systematic reviews and meta-analyses

Simply naming a review systematic does not imply that it is valid, even though systematic reviews are considered to be evidence of the highest level in the hierarchy of studies evaluating the effectiveness of interventions [10]. Readers need to be sure that the methodology used to synthesize relevant information was appropriate. This can be achieved by answering a few critical questions [61]:

1. Did the review have a predetermined protocol?
2. Was the question well formulated?
3. Did the review include appropriate type of studies?
4. Were all relevant studies identified using a comprehensive method?
5. Were the included studies assessed in terms of their validity?
6. Was the data abstracted appropriately from each study?
7. How was the information synthesized and was the synthesis appropriate?

##### Tools assessing the quality of systematic reviews and meta-analyses

A tool commonly used to critically appraise the quality of a systematic review (with or without meta-analysis) is the *Assessment of Multiple Systematic Reviews* (*AMSTAR*) which consists of an 11-item questionnaire assessing the presence of key methodological issues [62]. SIGN has developed, as well, different methodology checklists for the most common types of studies, including systematic reviews. This checklist covers all main topics of a systematic review (SR) aiming to facilitate the process of critical appraisal [46].

##### Tools assessing the reporting quality of systematic reviews and meta-analyses

The *Meta-analyses of Observational Studies in Epidemiology* (*MOOSE*) guidelines were designed to promote the reporting quality in reviews of observational studies [63]. The MOOSE checklist includes guidelines for the reporting of the background, search strategies, methods, results, discussion, and conclusion of the study [64]. The *Institute of Medicine* (*IOM*) *of the National Academies*, also, has developed standards for conducting systematic reviews in order to help authors improve the reporting quality of their studies [65]. The *Preferred Reporting Items for Systematic Reviews and Meta-Analyses* (*PRISMA*) *Statement* focuses on ways in which authors can ensure the transparent and complete reporting of systematic reviews and meta-analyses. PRISMA consists of a checklist of 27 items indispensable in the reporting of SRs and meta-analyses and a flow diagram illustrating the flow of information through the four different phases of a systematic review [66].

The main function of an abstract of a systematic review is to signal its systematic methodology. However, despite the development of PRISMA, which gave some guidance for abstracts, the quality of abstracts of systematic reviews, as in RCTs, still remained poor [67]. The PRISMA for Abstracts consists of a 12-item checklist of items to report when writing an abstract of a SR with or without meta-analyses (Table 4) [68].

**Table 4:** **The PRISMA for Abstracts checklist [** 68 **]**

**Item**	**Description**
Title	
1. Title	Identification of the study as a systematic review, meta-analysis, or both
Background	
2. Objectives	The research question including components such as participants, interventions, comparators, and outcomes
Methods	
3. Eligibility criteria	Study and report characteristics used as criteria for inclusion
4. Information sources	Key databases searched and search dates
5. Risk of bias	Methods of assessing risk of bias
Results	
6. Included studies	Number and type of included studies and participants and relevant characteristics of studies
7. Synthesis of results	Results for main outcomes (benefits and harms), preferably indicating the number of studies and participants for each. If meta-analysis was done, include summary measures and confidence intervals.
8. Description of the effect	Direction of the effect (i.e., which group is favored) and size of the effect in terms meaningful to clinicians and patients
Discussion	
9. Strengths and limitations of evidence	Brief summary of strengths and limitations of evidence (e.g., inconsistency, imprecision, indirectness, or risk of bias, other supporting or conflicting evidence)
10. Interpretation	General interpretation of the results and important implications
Other	
11. Funding	Primary source of funding for the review
12. Registration	Registration number and registry name

### Applying evidence into practice - introducing GRADE

Health-care professionals are urged to inform their clinical decisions with the best relevant evidence. Clinicians, thus, are expected not only to deliver a treatment but to choose the best treatment option based on their patients' needs and preferences while balancing all benefits and harms. However, translating research findings into clinical guidelines can often be challenging. Moreover, users of guidelines need to be confident that they can rely on existing recommendations. Grading of Recommendations Assessment, Development and Evaluation (GRADE) is a system for grading the quality of evidence in order to develop recommendations that are as evidence-based as possible [69]. The GRADE methodology consists of the formulation of a clear clinical question which is followed by the identification of all relevant outcomes from systematic reviews, rated depending on how important they are for the development of a recommendation [70]. Judgments about the quality of evidence for important outcomes are made, and specific recommendations are formulated based on the strength of evidence and net benefits [69]. GRADE is not only a rating system. It provides a structured process for developing the strength of recommendations, and its explicit and comprehensive approach ensures the transparency of the judgments made [71].

## Conclusions

Clinical decisions guided by valid and reliable research findings are considered to be more focused and effective than others [1]. Evidence indicates that controlled trials and systematic reviews with inadequate methodology are more susceptible to bias [6,14]. Optimal reporting of RCT and SR abstracts is particularly important in enabling increased and sufficient access to evidence. The development of standards for the reporting of scientific results is essential for dissemination of new knowledge [58].
